# Virtual reality in the treatment of eating disorders

**DOI:** 10.1002/cpp.2622

**Published:** 2021-06-05

**Authors:** Giuseppe Riva, Clelia Malighetti, Silvia Serino

**Affiliations:** ^1^ Applied Technology for Neuro‐Psychology Lab. Istituto Auxologico Italiano Milan Italy; ^2^ Humane Technology Lab. Università Cattolica del Sacro Cuore Milan Italy

**Keywords:** attentional biases, eating disorders, full body illusions, virtual reality, VR cue exposure, VR reference frame shifting

## Abstract

Over the last 25 years, virtual reality (VR) has offered innovative solutions for targeting different key symptoms of eating disorders: from craving to negative emotions, from attentional biases to body dissatisfaction. The present narrative review assesses the existing literature in these areas trying to identify their different levels of clinical evidence. Specifically, the review presents four clinical approaches based upon VR and their implications in the treatment of eating disorders: VR cue exposure, VR reference frame shifting, VR for correcting body distortions and attentional biases. In general, existing findings demonstrate the clinical value of VR. On one side, the present review suggests that two VR‐based techniques—VR exposure and reference frame shifting—have a significant research support and provide a possible advantage over traditional cognitive‐behavioural therapy (CBT) for bulimia nervosa and binge eating disorder. On the other side, two emerging VR applications—multisensory body illusions and the use of VR for the modification of attentional biases—even if supported by preliminary data still need further research.

**Key Practitioner Message**

- Over the last 25 years, virtual reality (VR)‐based applications have been used to target several core clinical aspects of EDs, such as binge eating, craving, anxiety, body distortion and attentional biases.
- In the present narrative review, we proposed a recent neuroscientific framework as an explanation of the rationale behind the clinical use of VR.
- We secondly review two VR‐based treatments—VR cue exposure and VR reference frame shifting—and discuss the results obtained by the available randomized clinical trials.
- We finally discuss the use of two emerging techniques—body illusions and VR for the modification of attentional biases—as promising therapeutic tools for ED patients.

## 1 INTRODUCTION

In general terms, virtual reality (VR) can be described as ‘an advanced form of human–computer interface that allows the user to interact with and become immersed in a computer‐generated environment in a naturalistic fashion’ (Schultheis & Rizzo, 2001) (p. 82). Key concepts related to the use and implementation of a VR set‐up are immersion and presence (Riva et al., 2003). Technologically speaking, immersion defines the degree (from non‐immersive to fully immersive) to which the user is isolated from the real world when interacting with digital environments. From a psychological perspective, however, the added value of VR over other synthetic experiences is the feeling of presence, that is, the sensation of actually ‘being there’ inside the digital environment that substitutes real perceptions (Riva, 2020). The feeling of presence offered by VR could be an incredible instrument for assessing and treating mental health disorders because individuals feel immersed and act in a digital world as if it were real, thus living meaningful experiences.

Over the last 25 years, one of the most effective clinical applications of VR is the development of innovative treatment approaches for patients with eating disorders (EDs) (Clus et al., 2018; Ferrer‐García et al., 2013; Ferrer‐García & Gutiérrez‐Maldonado, 2012; Gutiérrez‐Maldonado et al., 2016). EDs are mental illness that involved severe disturbances in eating behaviours and related thoughts and emotions (Malighetti et al., 2019). Anorexia nervosa (AN), bulimia nervosa (BN) and binge eating disorder (BED) are important examples of EDs that are recognized as having severe psychological and physiological consequences (APA, 2013).

AN is an ED characterized by severe underweight, accompanied by an intensive fear of gaining weight, a strict and restrictive diet and purging behaviour (self‐induced vomiting, laxative abuse, use of diuretics, etc.) (APA, 2013). The persistent lack of recognition of the severity of the current low body weight is one of the core features of AN, a disabling, deadly disorder with a high disease burden. BN is characterized by repeated episodes of overeating. In a short period of time patients will eat more food than others would eat in the same situation. Each episode is followed by actions to compensate for the large food intake and to avoid weight gain. The most common behaviour is to induce vomiting, but they can also abuse laxatives, fast for periods or take part in excessive amounts of exercise. BED is a severe ED characterized by recurrent episodes of eating large quantities of food, in a discrete period of time, coupled with a sense of loss control over one's eating and emotional distress (APA, 2013) These episodes are often followed by shame, abhorrence or depressive thoughts. The binge episodes that characterized BN and BED can be triggered by various emotional and environmental factors, such as a significant life event, often relating to loss, sexual conflict, or significant life changes that causes the individual to be self‐critical or experience negative affect, distress and loneliness (Burton & Abbott, 2017).

To achieve a better explanation of the clinical rationale behind the clinical use of VR in this population, a recent neuroscientific framework has been proposed. The starting point is the evidence that body representation disturbances are a clinical key feature of EDs (APA, 2013), involving different sensory modalities, from tactile perception (Keizer et al., 2011, 2012), proprioception (Carey & Preston, 2019; Case et al., 2012; Guardia et al., 2012; Scarpina et al., 2016), and interoception (Badoud & Tsakiris, 2017; Demartini et al., 2017; Di Lernia et al., 2019; Khalsa et al., 2015; Martin et al., 2019; Pollatos et al., 2016).

Globally, this growing literature supports the idea that a distorted multisensory integration may play a central role in EDs pathology (Riva & Dakanalis, 2018; Riva & Gaudio, 2018). Multisensory integration refers to how the brain integrates different streams of incoming information from different senses presented in the same space–time context into a coherent and uniform percept, namely the ‘body matrix’ (Moseley et al., 2012; Riva, 2018). The body matrix (Barrett, 2016) can be conceptualized as an internal model of the body that integrates input signals from different modalities, which are recalibrated according to predictions made through the stored conceptual (i.e., the meaning attributed to the body), perceptual (i.e., the size and the shape of the body), and episodic (i.e., the autobiographical events related to the experience of the body) (Blanke, 2012; Riva, 2018; Riva & Dakanalis, 2018; Tuena et al., 2017) information about the body.

In this view, the experience of the body is the result of a probabilistic process and may be not adhere to the characteristics of the physical body (Badoud & Tsakiris, 2017; Ferri et al., 2017). Recent studies (Matamala‐Gomez et al., 2021; Riva & Dakanalis, 2018; Riva & Gaudio, 2018) suggest that an impairment in this predictive process, such as altered feedback from and towards the body matrix, could be involved in the aetiology of EDs. Specifically, the multisensory body integration deficit may impair a patients' abilities (Riva & Dakanalis, 2018; Riva & Gaudio, 2018): (a) to recognize their relevant internal bodily signals that can predict potential emotional consequences and (b) to update their negative memories of body‐related events. The first impairment produces difficulties in identifying and using the appropriate strategies to modulate the emotional response, that could lead to behavioural control difficulties in stressful situation, as well as avoiding emotional eliciting situations (Caslini et al., 2016; Lavender et al., 2015). Accordingly, the first impairment can explain the food‐related and body‐related emotional disorders experienced by EDs patients, while the second one could explain the distortion in the experience and satisfaction with one's own body.

In the following paragraphs we will first review two VR‐based treatments—VR Cue exposure and VR reference frame shifting—that have received significant research support and could provide a possible advantage over traditional therapies. Finally, we will discuss the use of two emerging techniques—full bodily illusions and the modification of attentional biases—that, even if supported only by preliminary data, could be considered as promising therapeutic tools for EDs patients.

### 1.1 VR cue exposure

One of the most common clinical applications of VR in EDs is the cue exposure therapy (CET). In general terms, VR‐CET aims to extinguish/habituate craving (an intense and uncontrollable desire for a specific food) and anxiety responses to food‐related cues, and thus reducing the associated risk of overeating in patients with BN or BED (Ferrer‐García et al., 2019; Gutiérrez‐Maldonado, Pla‐Sanjuanelo, et al., 2016; Gutiérrez‐Maldonado, Wiederhold, et al., 2016; Koskina et al., 2013). This procedure is based on the classical conditioning model of binge eating: the gradual and repeated exposure to a binge cue aims at extinguishing the association between the cue (conditioned stimulus) and the maladaptive binge response (conditioned response). VR technology offered several advantages over other traditional exposure procedures (such as in vivo or imagery exposure) to help patients in reducing food craving and anxiety levels. Patients could enter safe and ecological simulations of the real‐life scenarios that more closely approximate the settings in which problematic eating behaviours usually take place. Moreover, VR permits the repeated delivery of the same cues/scenarios with a rich multisensory stimulation while allowing the response to attenuate, graded in difficulty, and customized for each specific patient. The possibility to successfully use virtual stimuli in cue exposure treatments has been proved as effective in different experimental studies. According to Gorini et al. (2010), virtual food was able to produce the same emotional reactions of real food in patients with EDs, and those reactions were also stronger than the one produced by photographs of food. Furthermore, Perpiñá et al. (2013) confirmed that after exposure to virtual food cues, ED patients experienced greater emotional involvement and dysphoria than controls; in contrast, controls only reported a high desire to eat during exposure to the virtual food.

Recently, a randomized controlled trial (Ferrer‐García et al., 2017) with a 6‐month follow‐up (Ferrer‐García et al., 2019) confirmed the validity of this approach in a sample of patients with BN and BED. The trial consisted in a randomized, parallel‐group study conducted at five ED European centres and involved a sample of 29 patients with BED and 35 patients with BN with no current comorbid severe mental disorders. The entire sample showed active episodes of binge eating after a first treatment level of CBT. After the unsuccessful CBT treatment, patients were randomly assigned to one of the two second‐level treatment conditions, six VR‐CET sessions or six additional CBT sessions (A‐CBT). The assessment was made in three times: at the end of the first treatment (CBT), at the end of the second‐level treatment and at a 6‐month follow‐up (Ferrer‐García et al., 2019). All randomized participants (N = 64) finished the second‐level treatments and completed the post‐treatment assessment, while only 58 patients completed the 6‐month follow‐up. Both second‐level treatment consisted in six twice‐a‐week sessions held over 3 weeks. The session was individual and 60 min long. The A‐CBT sessions aimed to reinforce behavioural improvements by practising the techniques learned during the initial organized CBT programme, with a focus on specific problem areas found by subjects and clinical psychologists at the end of their initial CBT course. Before starting the VR‐CET sessions, patients were assessed through a validated VR‐based cue exposure software which included a database of 30 virtual sweet and savoury foods, such as cake, pizza, chips, hot dog, and four every day real‐life environment where patients usually binge (kitchen, dining room, bedroom, and cafeteria) (Pla‐Sanjuanelo et al., 2016). Patients were asked to indicate the level of food craving elicited from 2D images of these foods and environments in order to construct an exposure hierarchy of situations for use in the VR‐CET sessions. Participants were subsequently exposed to a hierarchy of virtual environments that simulated various food‐related situations where they were exposed to the foods that they had previously rated as the ones that triggered the highest levels of craving from the previously described list of 30 items (Ferrer‐García et al., 2014; Ferrer‐García et al., 2019). The exposure to each hierarchy step ended when the participant's anxiety level (assessed on a visual analogue scale from 0 to 100 displayed on the laptop's monitor) decreased by 40 percent in relation to the level registered at the beginning of the exposure session or after 60 min of exposure. The results showed that both second‐level treatments improved patients' eating‐related anxiety, but with a better overall short‐term outcome and long‐term outcome (Ferrer‐García et al., 2019) in the VR‐CET group with significantly higher reduction in number of binge and purge episodes and self‐reported tendency to engage in episodes of overeating, food craving, and anxiety than the A‐CBT group.

Furthermore, there is evidence of positive effects of VR‐CET also with patient with AN (binge and purge type) and BN (Cardi et al., 2012; Roncero & Perpiñá, 2015). Two recent case reports showed lower levels of anxiety and avoidance behaviour related to food after VR‐CET. Specifically, the patient with AN increased BMI and decreased the binge/purge behaviours (Cardi et al., 2012), and the patient with BN eliminated binges and purges episodes (Roncero & Perpiñá, 2015). Moreover, VR exposure has facilitated the exploration of emotions and thoughts during the moment of eating the virtual food, enabling safe and therapeutic discussions in an ecological environment (Perpiñá & Roncero, 2016).

Finally, a recent meta‐analysis (Low et al., 2021) showed the efficacy of VR‐based intervention in reducing situation‐induced body dissatisfaction and frequency of binges, and highlighted the potential of VR in helping EDs develop coping strategies to food/situational triggers. Specifically, VR cue exposure resulted to be particularly effective in reducing food craving (an intense and uncontrollable desire for a specific food) and anxiety that typically trigger binge episodes in patients with BED and BN (Brown et al., 2020; Burton & Abbott, 2017; Butler & Heimberg, 2020).

### 1.2 Correcting multisensory integration deficits in ED: The reference frame shifting approach

To modify the stored negative memories of the body and reorganize patients' multisensory integration processes (Riva & Dakanalis, 2018), VR could be considered an emerging and promising approach (Matamala‐Gomez et al., 2018; Pedroli et al., 2018; Riva, 2011; Riva et al., 2018; Tuena et al., 2020). One of the most effective technique that could be implemented with the use of VR is the ‘reference frame shifting approach’ (Akhtar et al., 2017; Riva, 2011), that is focused on the reorganization of body‐related memories (Osimo et al., 2015; Riva, 2011). This approach involves the VR adaption of the imagery rescripting method, a therapeutic technique, used in the context of CBT, that aims at reducing distress associated with negative memories (Arntz & Weertman, 1999) and changing their meaning (Arntz, 2011). The rescripting can help the patient by offering a fresh perspective on events that happened in the past, eliciting new feelings, identifying unmet needs, and confronting the patient with reality so that a healthy mourning process can ensue. Previous studies indicated imagery as an effective intervention in decreasing negative self‐beliefs in patients with BN compared to a control condition that consisted of verbally examining the effects of beliefs on current functioning (Dugué et al., 2019). Imagery rescripting (ImRs) showed good outcomes in terms of short‐term decreased of binge eating and vomiting when conventional CBT had failed (Ohanian, 2002) as well as longer term maintenance (Wheatley et al., 2007). Riva et al. (2018) developed a specific body image rescripting protocol based on VR to enhance the CBT therapy. Specifically, this protocol consisted in a sensory training to ‘unlock’ the body memory by increasing the contribution of new egocentric somatosensory information related to the negative allocentric memory (Dakanalis et al., 2017; Riva et al., 2018). According to the Allocentric Lock Theory (ALT) (Riva & Dakanalis, 2018; Riva & Gaudio, 2018), indeed, individuals with EDs are locked in a body that they detest, which differs from the real one following an impaired ability to update a stored negative (offline/allocentric) representation of their own body with real‐time inputs (online/egocentric).

This altered body experience can be explained as the effect of a functional disconnection between top–down, premorbid learned tpredictions regarding the experience of the body and the processing of bottom–up perceptual information regarding its current state (Riva et al., 2018; Riva & Gaudio, 2012). This neuroscientific framework started from a consistent body of evidence indicating that our spatial experience, including the bodily one, involves the integration of different sensory inputs within two different spatial reference frames: egocentric and allocentric (Montana et al., 2019).

The egocentric parietal representations shape the short‐term memory of perceptual information, while the medial temporal allocentric representations sustain the long‐term memory (Byrne et al., 2007; Chirico et al., 2019; Cipolletta et al., 2017; Malighetti et al., 2016, 2019, 2020; Matamala‐Gomez et al., 2020; Serino et al., 2015, 2016; Tuena et al., 2021). Accordingly, these frames are supposed to play a critical role in the way memories are stored and retrieved (Akhtar et al., 2017; Byrne et al., 2007). Through the egocentric frame, we recall a memory from the original perspective (i.e., field mode), while using the allocentric frame, we retrieve the self‐engaged in the event as an object in the space (observer mode). In this view, the process of representing and retrieving events, including our body, is supported by the continuous ‘translation’ between the allocentric long‐term memory and the egocentric perceptual updating, which occurs via a coordinate transformation in the posterior parietal and retrosplenial cortices (Riva, 2014; Riva & Gaudio, 2012, 2018).

**FIGURE 1:**
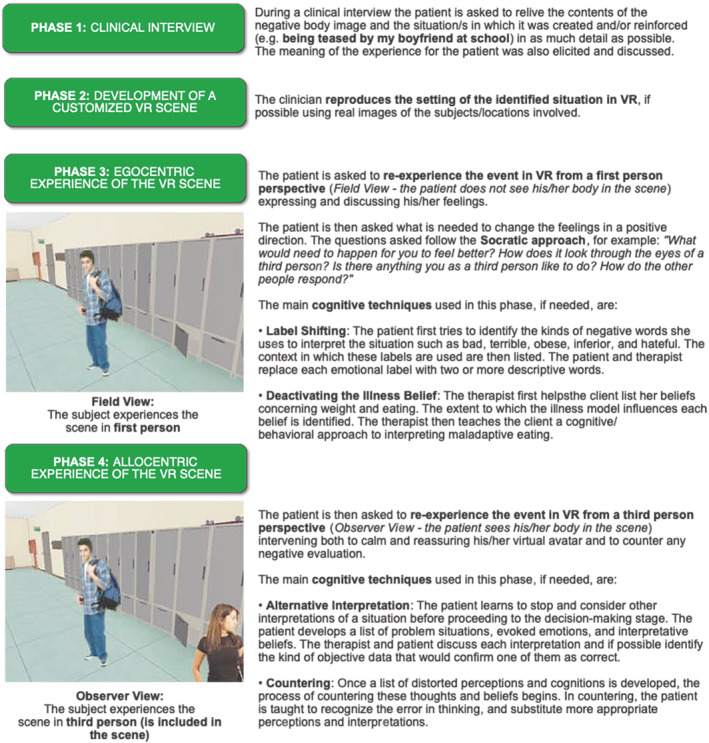
The four phases of the reference frame shifting approach [Colour figure can be viewed at wileyonlinelibrary.com]

VR sessions involved four phases (see Figure 1). The first dealt with the clinical interview between the clinician and patient to re‐experience in as much detail as possible the content of the negative allocentric body memory and the environment that triggered this content, for example, being teased by my boyfriend at school. During the interview, the therapist tried to explore the meaning of that experience for the patients. The second step consisted in the development of the customized VR scene. In this phase, the clinician recreated the same setting of the identified situation in VR. During the third and fourth phases the patients re‐experience the event in VR through an egocentric perspective and allocentric perspective respectively.

This approach has been successfully used as a randomized trials with BED patients (Cesa et al., 2013; Manzoni et al., 2016) and patients with BN (Varallo et al., 2021) allowing them to both update the contents of their body memory and improve the clinical outcomes over traditional CBT. Specifically, the first study involved 163 patients with BED (Manzoni et al., 2016) that were assigned to one of three treatment conditions: a standard behavioural inpatient programme (SBP), SBP plus standard CBT and SBP integrated with VR‐enhanced CBT. SBP was the common treatment condition for all participants and consisted in a 6‐week shelter. The treatment goal was to provide medical, nutritional, and psychological guidelines integrated with a low‐calorie diet and physical training. In the CBT treatment, patients were trained to self‐monitor their food intake and eating pattern thoughts, to identify problems in eating, mood and to develop alternative patterns. Finally, the third treatment condition consisted in VR‐enhanced CBT. After the first inpatients week, participants entered five weekly group sessions like the CBT ones (focused on concerns about body weight and shape and problematic eating) and 10 biweekly VR sessions. The VR treatment included 14 virtual environments that present critical situations related to the maintaining/relapse mechanisms and two body image comparison areas. Through the VR experience, patients practise both eating/emotional/relational management and general decision‐making and problem‐solving skills. By directly practising these skills within the VR environment, patients are helped in developing specific strategies for avoiding and/or coping with triggering situations (Manzoni et al., 2016). The study that involved BN patients (Varallo et al., 2021) allocated 24 patients to two conditions: the integrated multimodal medically managed inpatient programme (IP) and the same VR‐enhanced CBT described above. Both studies showed the effectiveness of VR‐CBT in decreasing the preoccupation with weight and fear of weight gain, reducing the episodes of binge eating and purging and increasing body satisfaction in BN and BED populations.

### 1.3 Innovative use of VR: Body illusions and modification of attentional biases

In the last decade, the growing research interest in the so‐called ‘bodily illusions’ based on multisensory integration processes and the rapid technological development of VR field has led to innovative VR‐based applications for evaluating and treating body distortions in ED. The possibility of using VR for correcting a dysfunctional body experience in this population started with the use of realistic ‘avatars’. Riva and his team pioneered the use of avatars to measure and treat body representation disturbances in patients with EDs with the development of the Body Image Virtual Reality Scale—BIVRS—(Riva et al., 1999). In this VR‐based task, patients were asked to select among nine avatars ranging from underweight to overweight and to indicate how they self‐perceived themselves. This virtual application was also used for treating body size distortions (Riva et al., 1998). In this VR‐based procedure, patients were first exposed to digitalized photographs of their real bodies in three different formats: underweight, normal weight, and overweight size. After the discussion of the feelings and emotions emerged in these phases with clinicians, patients were invited to model their perceived body image using an avatar and to compare it with both their actual body image and their ideal one.

The recent advances in the technological field offered the possibility of developing and testing more advanced VR‐based protocols (Riva, 2020). First, now it is possible to develop more realistic avatars presented both in first‐ and in third‐ person perspective, as suggested by the ALT theory (Riva & Gaudio, 2012). An example is offered by Monthuy‐Blanc et al. (2020), who developed the virtual immersive version of the Body Rating Scale (Stunkard et al., 1983). Participants were exposed to seven virtual bodies of increasing BMIs, from 15 to 33 kg/m^2^, created to match the number and the features of the original scale. The procedure consisted in asking participants to select the avatar that best represented their own body (i.e., perceived body size) and the one they wanted to have (i.e., ideal body size) in two different perspectives, namely the egocentric (first‐person perspective) and the allocentric perspective (third‐person perspective).

Second, the recent discoveries about the multisensory nature of body representations led to the development of innovative protocols, such as the ‘bodily illusions’. Starting from the pivotal work of Botvinick and Cohen (Botvinick & Cohen, 1998) with the well‐known ‘rubber hand illusion’ (RHI), multisensory bodily illusions have been used to investigate the plasticity of the bodily experience in both healthy and clinical populations (Bolognini et al., 2015; Costantini, 2014; Kilteni et al., 2015; Serino & Dakanalis, 2017). In the RHI, individuals see a touch on the artificial hand and, at the same time, perceive the touch on their real (hidden) hand; this multisensory stimulation can induce the illusionary transfer of the body ownership from the real hand to the artificial one.

Eshkevari et al. (2012) pioneered the use of the RHI paradigm in patients with EDs. Their findings demonstrated that patients experienced the illusion in a more intense way in comparison to healthy controls on both perceptual (i.e., proprioceptive drift) and subjective (i.e., self‐report questionnaires) level. Interestingly, this abnormal response to the illusion was found to correlate with their clinical symptoms. In the same perspective, Keizer and colleagues also showed that patients with AN experienced a stronger illusion when compared to healthy controls (Keizer et al., 2014). They also found that RHI was able to induce a decrease in the overestimation of hand width in patients with AN, which was not observed in healthy controls.

These findings have been interpreted in light of an increased sensitivity to visual inputs in EDs, which was able to override proprioceptive inputs. However, Mussap and Salton (2006) suggested that the greater sensitivity to the illusion might result from also from an ‘instability’ of the body internal model in ED, that would allow patients an easier incorporation of external objects (in this case, the rubber hand). More recently, an increasing body of pioneering studies revealed that it is also possible to use VR in these illusions (Maselli & Slater, 2013; Slater et al., 2009): individuals can experience the feeling of being the owner of a virtual body thanks to the delivery of a synchronous multisensory stimulation on the (hidden) actual body and its fake (virtual) counterpart.

Interestingly, literature focusing on the plasticity of body representations have found remarkable distortions in the bodily perception by experimentally changing the size of the artificial bodies. Participants perceive themselves significantly fatter or thinner than they really are, congruently with the embodied avatar (Normand et al., 2011; Piryankova et al., 2014; Preston & Ehrsson, 2014; Serino et al., 2016), with enormous clinical implications.

For instance, Serino et al. (2016) demonstrated the possibility to reduce body size distortions in a sample of female healthy participants thanks to the embodiment of a virtual skinny body. With the same set‐up, Keizer and colleagues (Keizer et al., 2016) showed that after the embodiment procedure, patients with AN exhibited a decrease in the overestimation of their shoulders, abdomen and hips, lasting for 2 h. A recent study demonstrated also the possibility of successfully integrating the use of the VR‐based embodiment procedure within a multidisciplinary treatment for AN as a potential useful instrument to monitor changes in multisensory bodily integration processes (Serino et al., 2019). A patient with a DSM‐5 diagnosis of AN underwent an intensive outpatient treatment. Three sessions of a VR‐based illusion were delivered. Results suggested that the responses to the VR‐based bodily illusion were able to effectively monitor changes in the multisensory bodily integration processes over these three times.

Finally, a systematic and hierarchical exposure to an embodied avatar has been also used by Porras‐Garcia et al. (2020) as a part of the standard CBT with a patient suffering from AN. The procedure consisted of five sessions in which the patient embodied an avatar of progressively increasing BMI. They found a decrease in the fear of gaining weight, body‐related anxiety, body image disturbances and body‐related attentional bias.

A further innovative advancement is the integration of eye‐tracking (ET) systems with the VR‐based embodiment techniques for evaluating and modulating attentional biases for ED patients.

Literature suggested the presence of specific attentional biases in patients suffering from ED. It is known that patients with EDs tended to devote more attentional resources to body‐related information in comparisons to other type of information (Rodgers & DuBois, 2016; Williamson et al., 2004). Thanks to the integration of ET systems with the VR‐based embodiment procedure, now it is possible to follow and record visual behaviour towards the body. This set‐up allows researchers and clinicians to collect objective parameters (such as fixations, the proportion of time spent observing each stimulus, the speed of orientation) to quantify the presence of attentional biases in both controlled and ecological environments. Porras‐Garcia et al. (2018) carried out two studies using a VR‐based embodiment procedure in which participants owned an avatar with their own body measurements, in comparison to a larger‐size one (Porras‐Garcia et al., 2019). They found that that women significantly devoted more attentional resources to weight‐related body parts than men.

## 2 CONCLUSION

The clinical use of VR with patients suffering from ED is based on theory‐driven psychological techniques grounded in cognitive science. Firstly, our review revealed the efficacy of VR‐CET in decreasing craving and anxiety responses to food. Several studies showed the efficacy of virtual food in producing the same emotional reactions and involvement of real food. Furthermore, recent RCTs confirmed the validity of this approach for patients with BN and BED in the long term. These findings showed not only an improvement related to the diagnostical dimensions involved in EDs in terms of binges and purges episodes but also an enhancement in the emotional reaction related to food in terms of anxiety and food craving. Finally, recent findings related to VR‐CET for AN patient confirmed the transdiagnostic nature of this clinical condition and the transversal nature of VR.

Secondly our review showed the efficacy of VR in modifying the experience of the body in EDs patients by changing the stored negative memories related to the body and thus reorganizing patients' multisensory integration processes. This approach involves the VR adaption of the imagery rescripting method that aims at reducing distress associated with negative memories (Arntz & Weertman, 1999) by changing their meaning (Arntz, 2011). The rescripting offers a fresh perspective on events that happened in the past and led to body dissatisfaction low self‐esteem or isolation, creating new feelings and meanings to store in memory. This approach has been successfully used as a randomized trials with BED patients (Cesa et al., 2013; Manzoni et al., 2016) and patients with BN (Varallo et al., 2021) allowing them to both update the contents of their body memory and improve the clinical outcomes over traditional CBT. Finally, besides the modification of allocentric memory of the body, our review showed that VR could be used to improve the processes of multisensory integration through multisensory body illusions and to reduce attentional biases to body‐related stimuli in EDs clinical populations.

However, despite the large amount of research developed so far and the opportunities it offers, the use of VR in routine clinical practice has not yet reached a high volume, at least in EDs treatment.

The first factor limiting the spread of VR technology is presence of difficulties related to the use of VR devices, that require specialized trainings usually not available in university courses. So, in the past, the centres that wanted to use this equipment in their daily clinical practice had to have, not only the financial resources for buying it, but also a budget dedicated to the hiring of technical personnel capable of implementing its installation and maintenance. Until very recently, the use of VR systems has involved the management of complex devices that require a certain level of technological knowledge and the assistance of technical staff. However, this limitation has also almost completely disappeared: the large‐scale commercialization of VR systems has brought down costs and exponentially improved the usability of the equipment.

The first generation of VR devices, between 1990 and 2015, was characterized by different technological issues, for example a low display resolution, a restricted field of view, and uncomfortable designs. In particular, the low display quality resulted in serious problems for the first VR users who reported constantly symptoms of cybersickness when interacting with the virtual environments. In addition, the first VR set‐up required expensive HMDs paired with equally expensive high‐end workstations to properly work (approximately 20.000/50.000 USD). Finally, developing and using a VR system required a significant technical knowledge that was typically available only in lab centres. All these issues necessarily limited the widespread adoption of VR in clinical settings. The year 2016 was a turning point in the VR world since the release of the first generation of HMDs targeted at consumers. The Oculus Rift—an HMD developed and manufactured by Oculus VR, a division of Facebook Inc., and sold only for 600 USD—introduced a new generation of more immersive and low‐cost devices for visualizing and interacting with virtual objects and environments (see Table 1).

**TABLE 1:** Commercially available VR devices

Mobility required	PC based			Mobile based			Console based	Standalone		
**System**	Oculus rift S	HTC cosmos/elite Vive pro/Pro eye	Valve index	Samsung gear VR	Google cardboard	Google daydream	PlayStation VR	HTC VIVE focus/plus	Oculus quest 2	Lenovo VR classroom 2
**Cost (USD)**	299	699/899 1199/1599	999	99	10–50	69–149	299	649/799	299	399
**Hardware requirements**	High‐end PC (>1000 USD)	High‐end PC (>1000 USD)	High‐end PC (>1000 USD)	High‐end Samsung phone (>600 USD)	Middle/high‐end Android phone or iPhone (>299 USD)	High‐end android phone (>499 USD)	PS4 (299 USD) or PS4 pro (399 USD)	None (internal snapdragon 835/XR2 processor)	None (internal snapdragon XR2 processor)	None (internal snapdragon 835 processor)
**Resolution**	2560 × 1440	2880 × 1660	2880 × 1660	2560 × 1440	Depends on the phone (minimum 1024x768)	Depends on the phone (minimum 1920x1080)	1920 × 1080	2880 × 1600	1832 × 1920 per eye	2160 × 1920
**Refresh rate**	80 Hz	90 Hz	120/144 Hz	60 Hz	60 Hz	90 Hz minimum	120 Hz	72 Hz	90 Hz	75 Hz
**Field of view**	115 degrees	110 degrees	130 degrees	101 degrees	From 70 degrees	96 degrees	100 degrees	110 degrees	100 degrees	110 degrees
**Body tracking**	High: Head tracking (rotation) and volumetric tracking (full room size—15 × 15 ft—movement)	High: Head tracking (rotation) and volumetric tracking (full room size—15 × 15 ft—movement)	High: Head tracking (rotation) and volumetric tracking (full room size—15 × 15 ft—movement)	Medium: Head tracking (rotation)	Medium: Head tracking (rotation)	Medium: Head tracking (rotation)	Medium/high: Head tracking (rotation) and positional tracking (forward/backward)	Medium/high: Head tracking (rotation) and volumetric tracking (full room size—15 × 15 ft—movement)	Medium/high: Head tracking (rotation) and volumetric tracking (full room size—15 × 15 ft—movement)	Medium/high: Head tracking (rotation)
**User interaction with VR**	High (using controllers)	Very high (using controllers and eye tracking)	High (using controllers)	Medium (using gaze, a built‐in pad or joystick)	Low (using gaze or a button)	Medium (using gaze or a joystick)	High (using a joystick or controllers)	Medium (using gaze, a built‐in pad or joystick)	High (using controllers or hand tracking)	Medium (using gaze, a built‐in pad or joystick)
**Software availability**	Oculus store	VIVE/steam store	Steam store	Oculus store	Google play or IOS store	Google play	PlayStation store	VIVE/steam store	Oculus store	Google play and Lenovo ThinkReality

In a few years, the cost required for installing and using complete VR system—including input, output, and 3D graphic computation—dropped by tens of thousands of dollars to just a few hundred. Other important developments include the expansion of faster computing power, the possibility to connect other advanced technologies (such Eye‐Tracker) interacting with VR for a more immersive, ecological and multisensory experience, and the possibility to develop virtual customized environments in a more easy and low‐cost way.

Regarding possible side effects caused using VR, the situation has also improved significantly. The most common one, as previously reported, is the phenomenon known as cybersickness (Regan & Price, 1994): the appearance of symptoms such as dizziness, nausea, fatigue, and disorientation, derived from the lack of consistency between the visually perceived movement and the information provided by the vestibular system (which, in contrast, detects the absence of movement) Although more research is needed on this phenomenon to further specify the mechanisms that produce it, some studies find that only a small percentage of patients who are exposed to virtual environments experience these negative effects, and that, when they appear, they do so for short periods and become less intense as immersion is repeated (Quintana et al., 2015).

Less favourable, unfortunately, is the situation related to the availability of content. As we have seen, the development of hardware has reached a level where neither the cost nor the difficulty of using the equipment represents an obstacle to its use as a resource in psychological assessment and treatment. However, there are almost no validated VR applications available commercially. In fact, all the VR apps discussed in this review have been developed by research teams and are not available for interested clinicians. So, now, all the interested clinicians have to develop personally their own VR software. This requires the use of computer programmes and programming languages that, currently, are only available to professionals trained to create 3D models, to animate objects and avatars, to assign lighting and textures, programme interactions, and so forth. In this view, the growth and improvement of commercially available VR clinical software is an essential condition for the disappearance of this significant barrier—probably the last one still standing—to the widespread use of VR technology in the treatment of EDs.

Overall, further research is needed to further clarify the efficacy VR‐based applications in the treatment of patients with EDs and their integration in standard therapies. Future RCTs should evaluate the efficacy of innovative VR‐based treatment, also including follow‐up evaluations.

## CONFLICT OF INTEREST

The authors declare no conflict of interest.

## Data Availability

Data sharing is not applicable to this article as no datasets were generated or analysed during the current study.
